# Predictive value of pretreatment PD-L1 expression in EGFR-mutant non-small cell lung cancer: a meta-analysis

**DOI:** 10.1186/s12957-021-02254-x

**Published:** 2021-05-08

**Authors:** Zhiyu Peng, Huahang Lin, Ke Zhou, Senyi Deng, Jiandong Mei

**Affiliations:** 1 Department of Thoracic Surgery, West China Hospital, Sichuan University, Chengdu, China; 2 Chest Oncology Institute, West China Hospital, Sichuan University, Chengdu, China; 3 Western China Collaborative Innovation Center for Early Diagnosis and Multidisciplinary Therapy of Lung Cancer, Sichuan University, Chengdu, China

**Keywords:** Non-small cell lung cancer, Epidermal growth factor receptor, Programmed death-ligand 1, Prognosis

## Abstract

**Objective:**

To investigate the predictive value of programmed death-ligand 1 (PD-L1) expression in non-small cell lung cancer (NSCLC) patients treated with epidermal growth factor receptor tyrosine kinase inhibitors (EGFR-TKIs).

**Methods:**

We conducted a systemic search of PubMed, EMBASE, and the Cochrane Library from 1 January 2000 to 30 August 2020, to identify related studies. We combined the hazard ratio (HR) and 95% confidence interval (CI) to assess the correlation of PD-L1 expression with progression-free survival (PFS) and overall survival (OS). We assessed the quality of the included studies by the Newcastle–Ottawa Scale (NOS). We performed subgroup analyses based on immunohistochemistry (IHC) scoring system, IHC antibodies, sample size, countries, and survival analysis mode. Sensitivity analysis and evaluation of publication bias were also performed.

**Results:**

Twelve studies including 991 patients met the criteria. The mean NOS score was 7.42 ± 1.19. Patients with high PD-L1 expression was associated with poorer PFS (HR = 1.90; 95% CI = 1.16–3.10; *P* = 0.011), while there was no association between PD-L1 expression and OS (HR = 1.19; 95% CI = 0.99–1.43; *P* = 0.070). Subgroup analysis prompted IHC scoring systems, IHC antibodies, and sample size have important effects on heterogeneity. The pooled results were robust according to the sensitivity analysis.

**Conclusions:**

The result of this meta-analysis suggested that PD-L1 expression might be a predictive biomarker for EGFR-mutant non-small cell lung cancer treated with EGFR-TKIs.

**Supplementary Information:**

The online version contains supplementary material available at 10.1186/s12957-021-02254-x.

## Background

Lung cancer is the major cause of cancer-related mortality among both men and women worldwide, and non-small cell lung cancer (NSCLC) accounts for approximately 85% of reported cases [1, 2]. Nearly 80% of NSCLC patients are diagnosed at the advanced stage, and the prognosis of patients with advanced stage NSCLC is extremely poor [3]. Epidermal growth factor receptor (EGFR) mutation is one of the most common driver oncogenes in NSCLC, and targeted therapy on EGFR-activating mutations has achieved great benefits [4, 5]. In the first line of treatment, several large-scale phase three trials have shown the better efficacy of EGFR tyrosine kinase inhibitors (EGFR-TKIs) to standard platinum-based chemotherapy [6–8]. However, nearly all the patients treated with EGFR-TKIs developed resistance after the early response [9, 10].

In the past few years, the immune checkpoint inhibitors (ICIs), which target the programmed death-1 (PD-1)/programmed death-ligand 1 (PD-L1) axis, have led to a long-lasting response in some patients with NSCLC by prompting the exhausted tumor infiltrating lymphocytes [11]. However, a limited effect of PD-1/PD-L1 inhibitors in patients with EGFR-mutant NSCLC was reported by Gainor et al. [12]. Moreover, the expression of PD-L1 was generally lower in EGFR-mutated tumors than in EGFR wild-type tumors. This might be the reason for the poor response to immune checkpoint inhibitors in EGFR-mutated tumors [12, 13]. The expression of PD-L1 reveals the immunogenic nature of the tumor microenvironment. Therefore, it is probably related to clinical outcomes of the treatments other than ICIs. At present, several studies have investigated the predictive value of PD-L1 expression in EGFR-mutant NSCLC patients treated with EGFR-TKIs [14–25]. However, previous studies regarding this topic have yielded conflicting results because of different sample size, antibody clone for immunohistochemistry (IHC), and IHC scoring system applied. A meta-analysis study on this issue has also been performed in the past [26]. However, this study included fewer studies and did not conduct a subgroup analysis looking for possible reasons for the large heterogeneity of results in previous studies. Therefore, we designed and performed the current meta-analysis to further determine the predictive value of PD-L1 expression in NSCLC patients treated with EGFR-TKIs.

## Methods

A meta-analysis does not require necessary patients’ consent or approval. We carried out this meta-analysis in accordance with the Preferred Reporting Items for Systematic Reviews and Meta-Analyses (PRISMA) statement (Supplementary Table S1) [27]. The protocol was registered in International Platform of Registered Systematic Review and Meta-analysis Protocols (INPLASY202140093).

### Literature searching strategy

From the establishment date of databases to 30 August 2020, we searched PubMed, Embase, Cochrane library and China National Knowledge Infrastructure (CNKI) with terms related to “non-small cell lung cancer,” “PD-L1,” “EGFR-TKIs,” and “prognosis.” The detailed search strategy for each database is presented in Supplementary Table S2. Besides, potentially eligible studies were also manually checked through the reference lists of included studies.

### Inclusion and exclusion criteria

The inclusion criteria were as follows: (1) patients were diagnosed with advanced NSCLC and treated with EGFR-TKIs alone; (2) the primary outcomes were progression-free survival (PFS) and/or overall survival (OS); (3) the relationship between PD-L1 expression and PFS/OS was described; (4) necessary survival data including hazard ratio (HR), 95% confidence interval (CI), or Kaplan-Meier survival curve was provided.

The exclusion criteria were (1) a previous history of chemotherapy or radiotherapy; (2) case reports, comments, corresponding letters, reviews, and meeting abstracts; (3) necessary survival data to calculate the HR with 95% CI was not provided.

### Data extraction and assessment of the study quality

Each study was reviewed and evaluated by two independent researchers (ZYP and HHL). Disagreement was resolved through discussion with a third researcher (JDM). The following data were recorded: the name of the first author, publication year, origin of the study, study period, sample size, type of cancer, stage of cancer, detective methods, and grouping methods. The primary outcomes were the hazard ratio (HR) and 95% confidence interval (CI). Where necessary, we applied Engauge Digitizer (version 4.1; http://digitizer.sourceforge.net) to extract the HR and 95% CI from survival curves [28]. According to the method described by Afzal, HR of the lowest versus the highest level of PD-L1 expression was extracted when the levels of PD-L1 expression were divided into several groups [29]. The quality of included studies was assessed by the Newcastle–Ottawa Scale (NOS). NOS scores of ≥ 6 were identified as high-quality studies.

### Statistical analysis

Stata (version 16.0; Stata Corporation, TX, USA) software was applied to analyze the extracted data. HR with 95% CI was used to assess the significance of PD-L1 expression on OS and PFS of the patients with NSCLC treated with EGFR-TKIs. *I*-square (*I*^2^) test was applied to assess the heterogeneity among the studies. Statistical significance was set at a *P* value of 0.05. A fixed-effect model was applied when the heterogeneity was considered to be insignificant (*I*^2^ < 50%), or else, a random effect model was applied. In addition, we performed a sensitivity analysis by removing included studies one-by-one to test whether the results were robust. We applied Egger’s test and Begg’s test to assess the possibility of publication bias.

## Results

### Characteristics of studies

**Fig. 1:**
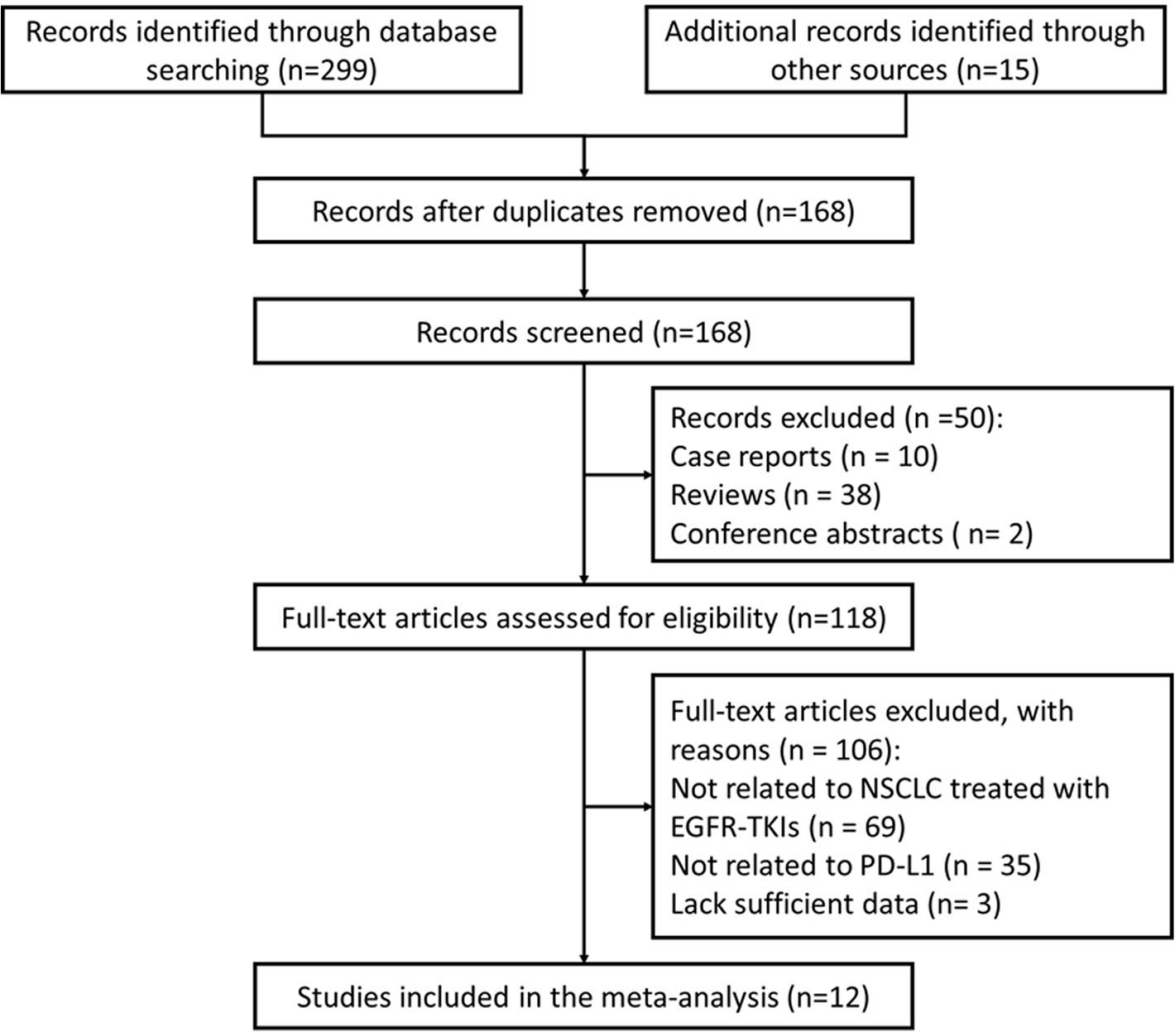
Flow diagram of studies retrieved, screened, and selected for further analysis

The flowchart of the study selection is shown in Fig. 1. During the primary search, we retrieved a total of 299 studies. After removing duplicates and screening the titles and abstracts, 118 studies were selected for full review. Full texts of 118 candidate studies were carefully reviewed and 106 of them were excluded (Fig. 1). Eventually, we included 12 original studies published between 2015 and 2020 in this meta-analysis. The characteristics of all eligible studies are presented in Table 1. The mean NOS score of these included studies was 7.42 ± 1.19.

**Table 1:** Characteristics of qualified records in meta-analysis

Author	Year	Region	Study period	Cases	Cancer type	Stage	Detective method	Cut-off value	Outcomes	EGFR-TKIs	NOS scores^a^
Yoon B. W [26].	2020	Korea	2014.1–2019.12	131	NSCLC	III–IV	IHC (22C3)	TPS: 1%, 50%	PFS, OS	Gefitinib, Erlotinib, and Afatinib	9
Yang C. Y [24].	2020	China	2012–2017	153	ADC	IIIB–IV	IHC (22C3)	TPS: 1%, 50%	PFS	Gefitinib, Erlotinib, and Afatinib	9
Hsu K. H [22].	2019	Korea	2012–2017	123	ADC	IIIB–IV	IHC (SP263)	TPS: 1%, 25%, 50%	PFS, OS	Gefitinib, Erlotinib, and Afatinib	8
Matsumoto Y [23].	2019	Japan	2013.8–2017.12	52	NSCLC	III–IV	IHC (28-8)	TPS: 50%	PFS	Gefitinib, Erlotinib, Afatinib, Gefitinib + Erlotinib, Afatinib + Gefitinib and Erlotinib + Afatinib	8
Kobayashi K [19].	2018	Japan	2001–2009	32	NSCLC	NA	IHC (SP263)	5% of tumor cells	PFS, OS	Gefitinib and Erlotinib	7
Su S [20].	2018	China	2016.4–2017.12	84	NSCLC	IV	IHC (SP142)	TC3/IC3, TC1-2/IC1-2, and TC0/IC0	PFS	Gefitinib	7
Yoneshima Y [21].	2018	Japan	2013.1–2017.12	80	ADC	III–IV	IHC (22C3)	TPS: 1%, 50%	PFS	NA	7
Bai Y. C [18].	2018	China	2011–2015	73	NSCLC	IIIB–IV	IHC (E1L3N)	Moderate staining: 5%	OS	NA	6
Soo R. A [17].	2017	Korea	2011–2014	90	NSCLC	III–IV	IHC (SP142)	H score^b^: 109.23(mean)	PFS, OS	Gefitinib, Erlotinib, and Dacomitinib	7
Tang Y [16].	2015	China	2008.1–2014.3	89	NSCLC	IIIB–IV	IHC (E1L3N)	H score^b^: 5	PFS, OS	1st TKIs and 2nd TKIs	8
Lin C [15].	2015	China	2010.4–2014.7	56	ADC	III–IV	IHC (ab58810)	H score^b^: mean of all patients	PFS, OS	Gefitinib and Erlotinib	6
D’ Incecco A [14].	2015	Italy	NA	95	NSCLC	III–IV	IHC (ab58810)	Moderate staining: 5%	OS	Gefitinib and Erlotinib	6

*NA* Not available, *ADC* Adenocarcinoma, *NSCLC* Non-small cell lung cancer, *IHC* Immunohistochemistry, *TPS* Tumor proportion score, *TC* Tumor cell, *PFS* Progression-free survival, *OS* Overall survival, *NOS* Newcastle–Ottawa Scale

^a^0–3 points: low quality, 4–6 points: moderate quality, 7–9 points: high quality

^b^H score is defined as the percentage of positively stained tumor cells multiplied by the intensity of staining

### Association between PD-L1 expression and survival outcomes

**Fig. 2:**
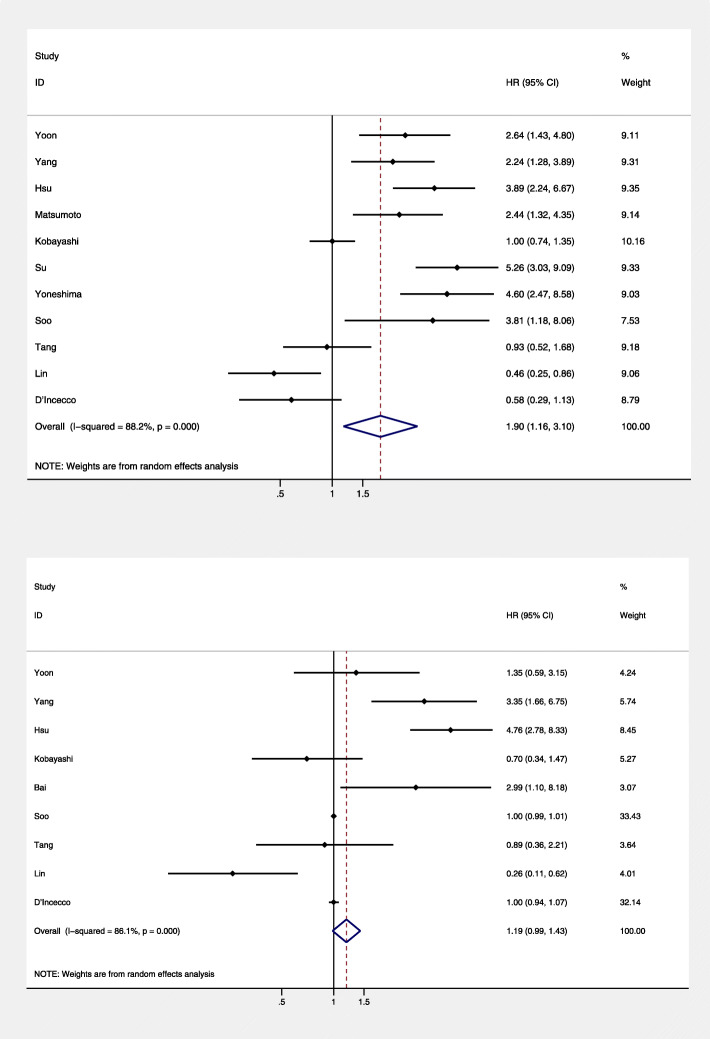
Forest plot of the association between PD-L1 expression and **a** progression-free survival and **b** overall survival. CI, confidence interval; HR, hazard ratio

A total of 12 studies involving 991 patients were included in this meta-analysis. The pooled results showed that higher PD-L1 expression was associated with poorer PFS (HR = 1.90; 95% CI = 1.16–3.10; *P* = 0.011), but with high heterogeneity (*I*^2^ = 88.2, *P* = 0.000) (Fig. 2a). However, the level of PD-L1 expression was not associated with OS (HR = 1.19; 95% CI = 0.99–1.43; *P* = 0.070) (Fig. 2b) in NSCLC patients treated with EGFR-TKIs.

### Subgroup analysis

**Fig. 3:**
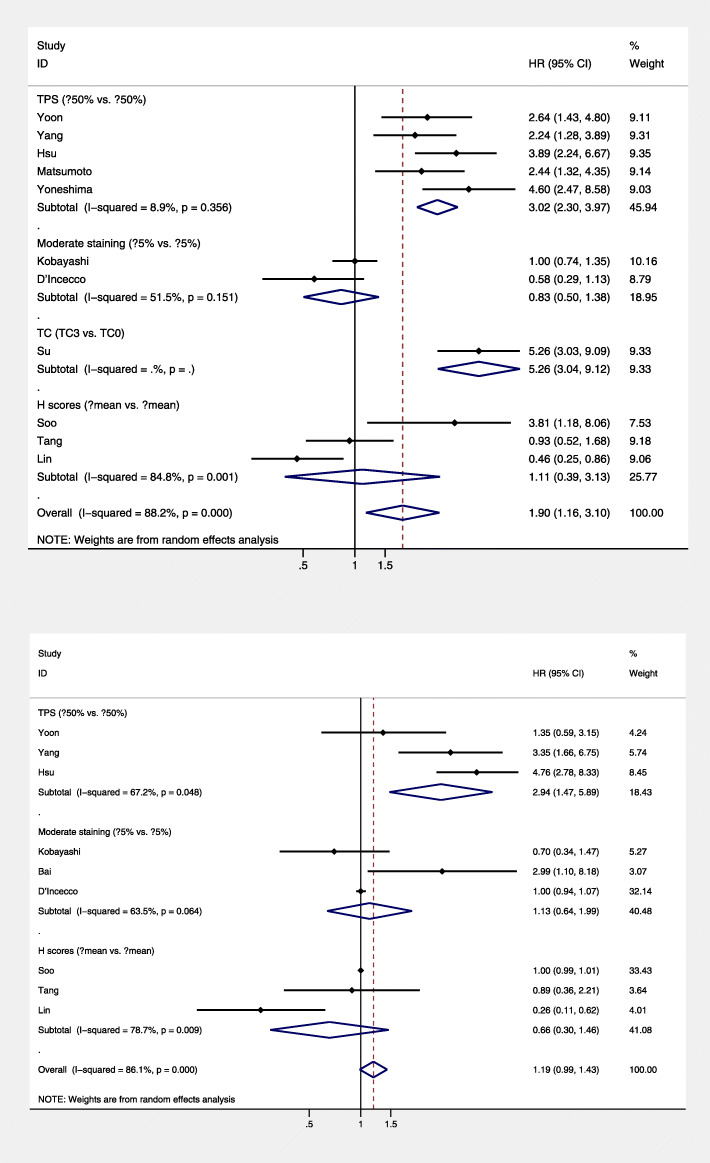
Subgroup analysis based on different scoring systems. **a** Stratified by the scoring system of PD-L1 with progression-free survival and **b** Stratified by the scoring system of PD-L1 expression with overall survival. CI, confidence interval; HR, hazard ratio; TPS, tumor proportional score; TC, tumor cells; H score is defined as the percentage of positively stained tumor cells multiplied by the intensity of staining

**Fig. 4:**
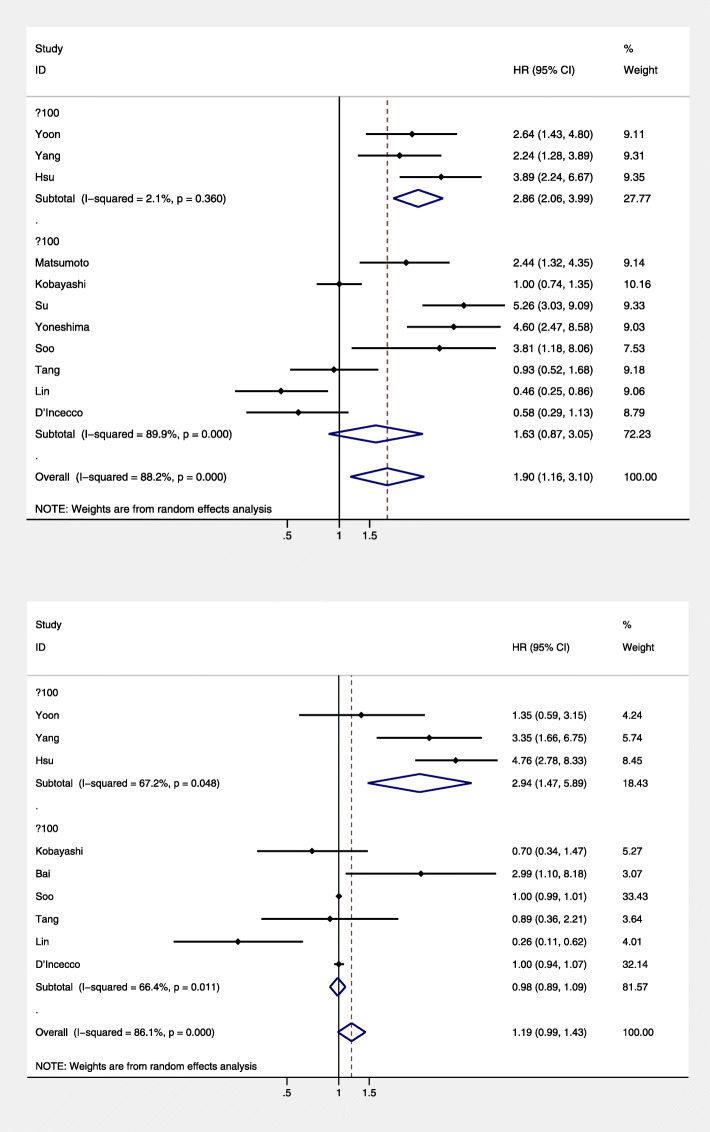
Subgroup analysis based on different sample size. **a** Stratified by the sample size of included studies with progression-free survival and **b** stratified by the sample size of included studies with overall survival. CI, confidence interval; HR, hazard ratio

**Fig. 5:**
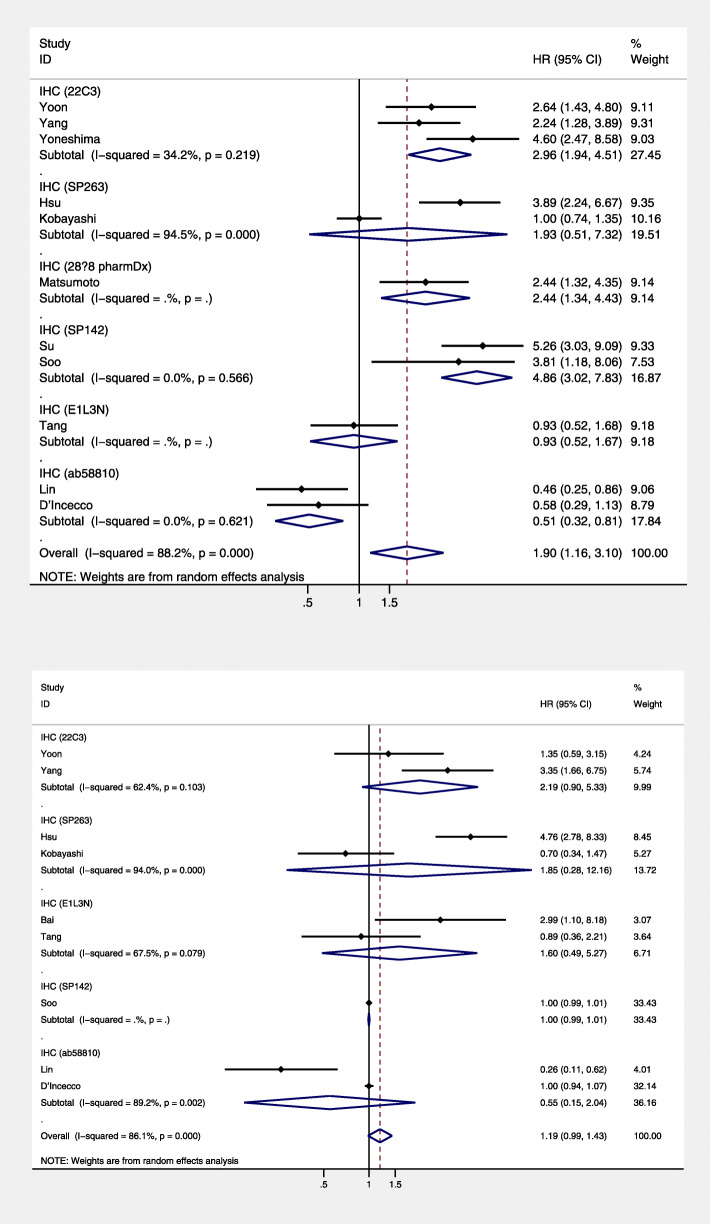
Subgroup analysis based on different IHC antibodies. **a** Stratified by the IHC antibodies of PD-L1 with progression-free survival and **b** stratified by the IHC antibodies of PD-L1 expression with overall survival. IHC, immunohistochemistry; CI, confidence interval; HR, hazard ratio

To reveal the potential heterogeneity, subgroup analysis stratified by IHC scoring systems, sample size, IHC antibodies, countries, and survival analysis mode was applied. The results of subgroup analysis indicated that patients with higher PD-L1 expression had poorer PFS in the following subgroups: tumor proportional score (TPS) of scoring system subgroup (HR = 3.12; 95% CI = 1.28–3.89; *P* = 0.000; Fig. 3a), sample size larger than 100 patients (HR = 2.96; 95% CI = 1.72–5.09; *P* = 0.000; Fig. 4a), and 22C3 IHC antibodies (HR = 2.96; 95% CI = 1.94–4.1; *P* = 0.000; Fig. 5a). As for OS, the results of subgroup analysis indicated that patients with higher PD-L1 expression had poorer OS in the following subgroups: TPS scoring system subgroup (HR = 4.17; 95% CI = 2.70–6.42; *P* = 0.000; Fig. 3b), sample size larger than 100 group (HR = 4.17; 95% CI = 2.70–6.42; *P* = 0.000; Fig. 4b). The results of the subgroup analysis revealed IHC scoring systems, IHC antibodies, and sample size may contribute to the heterogeneity (Fig. 5). The detailed results of the subgroup analysis were summarized in Table 2.

**Table 2:** Pooled HR of PD-L1 expression (high vs. low level) for OS and PFS according to subgroup analyses

Outcomes	Study number	Model	Test of association		Heterogeneity	
			HR (95% CI)	*P* value	I^2^ (%)	P-H
**PFS**	11	Random	1.897 (1.160, 3.104)	0.011	88.2	0.000
IHC scoring systems						
TPS (≥ 50% vs. < 50%)	5	Random	**3.020 (2.298, 3.968)**	**0.000**	8.9	0.356
Moderate staining (≥ 5% vs. < 5%)	2	Random	0.832 (0.503, 1.379)	0.476	51.5	0.151
TC (TC3 vs. TC0)	1	Random	5.263 (3.308, 9.116)	0.000	/	/
H scores^a^ (≥mean vs. <mean)	3	Random	1.106 (0.391, 3.130)	0.850	84.8	0.001
Sample size						
> 100	3	Random	**2.864 (2.058, 3.987)**	**0.000**	2.1	0.360
< 100	8	Random	1.628 (0.869, 3.049)	0.128	89.9	0.000
IHC antibodies						
22C3	3	Random	**2.956 (1.938, 4.510)**	0.000	34.2	0.219
SP263	2	Random	1.933 (0.511, 7.315)	0.331	94.5	0.000
28-8	1	Random	2.439 (1.342, 4.433)	0.003	/	/
SP142	2	Random	**4.858 (3.016, 7.827)**	0.000	0.0	0.566
E1L3N	1	Random	0.930 (0.517, 1.672)	0.808	/	/
ab58810	2	Random	0.511 (0.323, 0.807)	0.004	0.0	0.621
Survival analysis mode						
Multivariate analysis	6	Random	1.844 (0.911, 3.733)	0.089	86.4	0.000
Univariate analysis	5	Random	1.964 (0.895, 4.308)	0.092	91.7	0.000
Countries						
China	4	Random	1.508 (0.538, 4.224)	0.434	92.2	0.000
Italy	1	Random	0.580 (0.294, 1.130)	0.116	/	/
Japan	3	Random	2.173 (0.837, 8.580)	0.111	90.9	0.000
Korea	3	Random	3.346 (2.303, 4.860)	0.000	0.0	0.622
**OS**	9	Random	1.186 (0.986, 1.427)	0.070	86.1	0.000
IHC scoring systems						
TPS (≥ 50% vs. < 50%)	3	Random	**2.945 (1.471, 5.894)**	**0.002**	67.2	0.048
Moderate staining (≥ 5% vs. < 5%)	2	Random	1.127 (0.637, 1.994)	0.682	63.5	0.064
H scores^a^ (≥mean vs. <mean)	3	Random	0.657 (0.296, 1.458)	0.302	78.7	0.009
Sample size						
> 100	3	Random	**2.945 (1.471, 5.894)**	**0.002**	67.2	0.048
< 100	6	Random	0.985 (0.888, 1.093)	0.774	66.4	0.011
IHC antibodies						
22C3	2	Random	2.191 (0.901, 5.330)	0.084	62.4	0.103
SP263	2	Random	1.855 (0.283, 12.157)	0.520	94.0	0.000
E1L3N	2	Random	1.601 (0.487, 5.266)	0.439	67.5	0.079
SP142	1	Random	1.001 (0.991, 1.012)	0.852	/	/
ab58810	2	Random	0.548 (0.147, 2.036)	0.369	89.2	0.002
Survival analysis mode						
Multivariate analysis	6	Random	1.201 (0.668, 2.160)	0.540	80.7	0.000
Univariate analysis	3	Random	1.499 (0.986, 1.427)	0.441	93.7	0.000
Countries						
China	4	Random	1.238 (0.370, 4.145)	0.730	87.3	0.000
Italy	1	Random	1.000 (0.986, 1.427)	1.000	/	/
Japan	1	Random	0.699 (0.334, 1.463)	0.342	/	/
Korea	3	Random	1.884 (0.634, 5.361)	0.261	93.6	0.000

*TPS* Tumor proportion score, *TC* Tumor cell, *PFS* Progression-free survival, *OS* Overall survival

^a^H score is defined as the percentage of positively stained tumor cells multiplied by the intensity of staining

### Sensitivity analysis

**Fig. 6:**
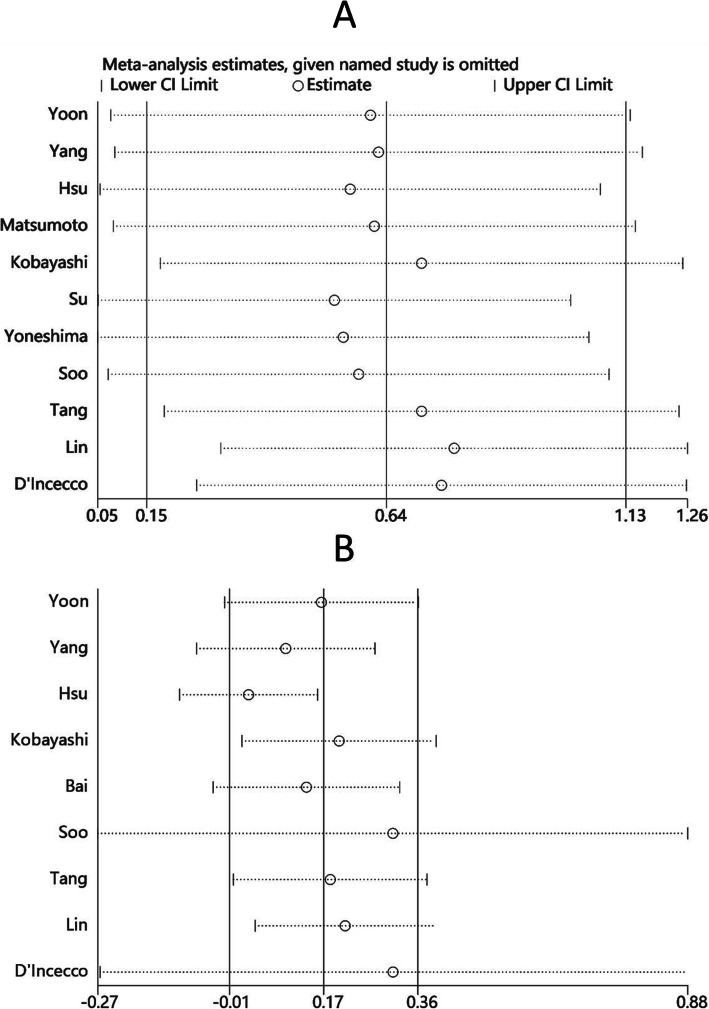
Sensitivity analysis of the association between pretreatment PD-L1 expression and PFS (**a**) or OS (**b**)

The results of the sensitivity analysis of OS and PFS were shown in Fig. 6. A sensitivity analysis was conducted by excluding each study from the meta-analysis at each time. The statistical results were steady, which indicated that the pooled results were robust.

### Publication bias

**Fig. 7:**
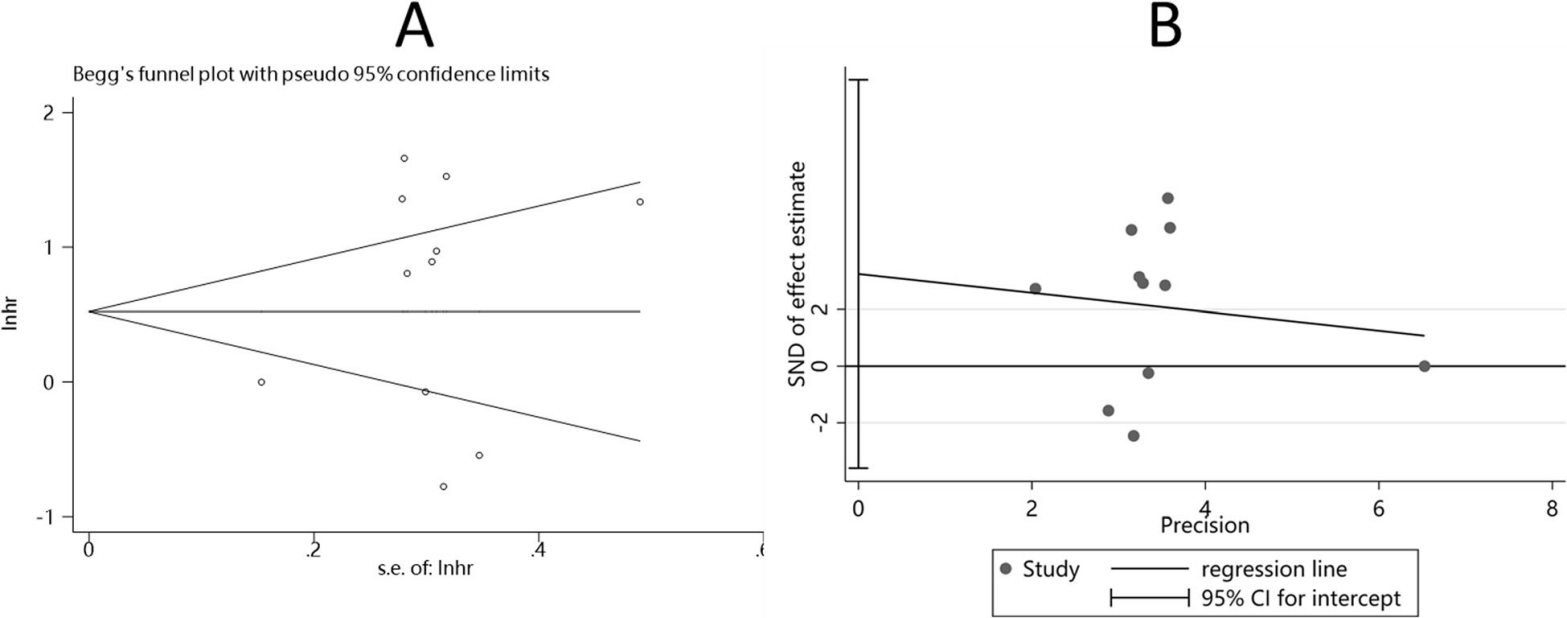
Begg’s funnel plot and Egger’s funnel plot showed no publication bias among the included studies. **a** Begg’s funnel plot of PD-L1 expression and PFS (*P* = 0.929). **b** Egger’s funnel plot of PD-L1 expression and PFS (*P* = 0.174)

The Begg’s funnel plot (*P* = 0.929) (Fig. 7a) was symmetric, and the *P* value of Egger’s test was 0.174 (Fig. 7b), which both indicated no significant publication bias. The existence of bias among studies about OS was not estimated because of the insufficient research number (less than 10).

## Discussion

Given that immune mechanisms are involved in EGFR-TKI resistance and that increased PD-L1 expression has been detected in the context of acquired resistance to EGFR-TKIs [30, 31], it is assumed that PD-L1 expression on tumor and immune cells may predict poor response to EGFR-TKI treatment in NSCLC patients with EGFR mutation. However, previous studies regarding this topic have not come to a unanimous conclusion [14, 19, 24]. Therefore, the present meta-analysis aimed to determine the predictive value of PD-L1 expression in NSCLC patients treated with EGFR-TKIs. In this meta-analysis, we found that NSCLC patients with higher PD-L1 expression were associated with poorer survival regarding PFS. However, there was no significant association between PD-L1 expression and OS, which suggested that the predictive value of pretreatment PD-L1 expression in non-small cell lung cancer treated with EGFR-TKIs needed to be further studied.

The subgroup analysis showed that higher PD-L1 expression was related to poor PFS in NSCLC patients treated with EGFR-TKIs. However, the PFS between high and low PD-L1 expression groups was similar in studies using different IHC scoring systems including moderate staining and H score. One of the possible reasons may be the insufficient sample size in the specific subgroup. In the subgroup analysis stratified by IHC scoring systems including TPS, moderate staining, tumor cells (TC), and H score, only TPS showed a robust result with low heterogeneity. Therefore, more studies should be performed to determine one reliable IHC scoring system and verify the results.

The use of different IHC antibodies, IHC scoring systems, and cutoff values may also affect the results and conclusions. This could explain why the existing data are ambiguous concerning whether tumor PD-L1 expression can predict the response to EGFR-TKI treatment. Six IHC antibodies (22C3 [21, 24, 25], SP263 [22], 28-8 [23], SP142 [17, 20], E1L3N [16, 18], and ab5880 [14, 15]) were used for the detection of PD-L1 expression and almost all the included studies used different antibodies. The subgroup analysis indicated that patients with higher PD-L1 expression had poorer PFS in the group of 22C3 IHC antibodies (HR = 2.96; 95% CI = 1.94–4.1; *P* = 0.000; Fig. 5a) and SP142 (HR = 4.86; 95% CI = 3.02–7.83; *P* = 0.000; Fig. 5a). Considering that 22C3 was the only approved companion diagnostic test for immune checkpoint inhibitors in lung cancer [32], it may be treated as a standard IHC antibody in the future. Given the inconsistent findings regarding the relationship between PD-L1 expression and responses to EGFR-TKIs, further validation should be conducted using standardized methods.

The use of different EGFR-TKIs may also be a reason for the inconsistent results. The detailed report of different kinds of EGFR-TKIs of the included studies was listed in Table 1. As we can see, most of the studies used different EGFR-TKIs, but few of them compared the predictive value of pretreatment PD-L1 expression in NSCLC with different EGFR-TKIs. Different kinds of EGFR-TKIs had various therapeutic effects and eventually led to different survival data. For example, the third generation TKI can overcome the resistance caused by T790M mutation and have better survival time than that of the first generation [33–35]. Therefore, different kinds of EGFR-TKIs should be considered as a mixed factor in future studies.

Several recent studies indicated that there were some mechanisms by which PD-L1 expression affects the prognosis of NSCLC patients treated with EGFR-TKIs. It has been reported that EGFR mutant NSCLC was more likely to exhibit an uninflamed phenotype with less immune cell infiltration, which suggested a lower likelihood of adaptive PD-L1 expression [36]. Therefore, higher PD-L1 expression in EGFR-mutated NSCLC might relate to the activation of oncogenes other than EGFR [37], which could lead to TKI resistance through bypass activation. On the other hand, immune cells within the tumor microenvironment may also account for EGFR-TKI resistance [24]. Therefore, we speculate that higher PD-L1 expression in NSCLC patients may lead to EGFR-TKI resistance through tumor microenvironment and eventually result in a poor prognosis for TKI treatment.

The sample size of patients included in a few studies was relatively small, and there was no prospective study in the present meta-analysis. A larger-scale, multicenter, prospective study focusing on the predictive value of PD-L1 expression in NSCLC patients treated with EGFR-TKI is encouraged in the future. Moreover, future studies could also attempt to explore the predictive value of pretreatment PD-L1 expression in NSCLC treated with EGFR-TKIs by different IHC antibodies, IHC scoring systems, and EGFR-TKIs.

Inevitably, the present meta-analysis had several potential limitations. First, although the search process was not restricted to languages, we searched only one native Chinese database in addition to the three general English databases, possibly ignoring studies from native databases in other languages. Second, some of the HRs with 95% CIs were evaluated using the Kaplan-Meier survival curves, which might be different from the original data. Third, the heterogeneity tests were significant in some of the pooled HRs of PFS and OS. The potential cause to explain the heterogeneity included the antibodies or IHC scoring systems used in different studies, the type of EGFR-TKIs, origin of the patients, and potential publication bias. Fourth, all the included studies were retrospective studies with relatively small sample size.

## Conclusions

In conclusion, this meta-analysis demonstrated that PD-L1 expression might be a negative predictive biomarker for EGFR-mutant NSCLC patients treated with EGFR-TKIs. Further study with standardized detection antibody, IHC scoring system, and larger sample size is warranted to validate the conclusion.

## Supplementary Information

**Additional file 1: Table S1**. Checklist of the PRISMA extension for meta-analysis. **Table S2**. Literature search criteria.

## Data Availability

All the data used in this study can be obtained from the original articles.

## Abbreviations

PD-L1: Programmed death-ligand 1
NSCLC: Non-small cell lung cancer
EGFR-TKIs: Epidermal growth factor receptor tyrosine kinase inhibitors
ICIs: Immune checkpoint inhibitors
HR: Hazard ratio
CI: Confidence interval
PFS: Progression-free survival
OS: Overall survival
NOS: Newcastle–Ottawa scalefvalue
IHC: Immunohistochemistry
EGFR: Epidermal growth factor receptor
PD-1: Programmed death-1
TPS: Tumor proportional score
TC: Tumor cell
